# Two new species and a new combination of *Allacta* (Blattodea, Ectobiidae, Pseudophyllodromiinae) from China, with notes on their behavior in nature

**DOI:** 10.3897/zookeys.836.31673

**Published:** 2019-04-08

**Authors:** Jia-Jun He, Yu-Hong Zheng, Lu Qiu, Yan-Li Che, Zong-Qing Wang

**Affiliations:** 1 Institute of Entomology, College of Plant Protection, Southwest University, Beibei, Chongqing, 400716, China Southwest University Chongqing China

**Keywords:** Dictyoptera, Blattaria, *
Temnopteryx
*, cockroaches

## Abstract

Two new species, *Allactabruna***sp. n.** and *Allactaalba***sp. n.**, from China are described and illustrated. *Allactahainanensis* (Liu et al., 2017), **comb. n.** is proposed and re-described; figures including genitalia are provided. A key is provided to all species from China based on males. Notes on the bionomics of this genus in China are provided.

## Introduction

The genus *Allacta* is widely distributed from southwestern China to northeastern Australia. It consists of 41 known species worldwide, five of which were known from China before this study. Wang et al. (2014) described the species *Allactaxizangensis*, re-described four other known species from China, and distinguished *Allacta* from related genera.

Liu et al. (2017) described a species *Temnopteryxhainanensis* from Hainan, China, based on morphological characters (without providing male genital characters), and which has reduced tegmina and wings. However, Princis (1963) proposed *Temnopteryx* is endemic to South Africa. Princis also established the generic diagnosis as follows: tegmina absent or shortened, hind wing reduced to lateral lobes or absent; tarsal claws symmetrical and unspecialized; and abdominal terga unspecialized. Fortunately, we obtained specimens of *T.hainanensis* from Hainan Province as well as a photograph of the holotype. We also collected specimens from Zhejiang and Hainan, among which two new species of *Allacta* were discovered, and we use this opportunity to describe them together. With an increase in field surveys, it has been possible to better observe the behavior in nature of *Allacta* species and the members of this genus are commonly found to be tree climbers in the forests.

## Materials and methods

Terminology used mainly follows Roth (2003). Genitalia terms are according to McKittrick (1964). Venation terms follow Li et al. (2018). Vein abbreviations in this article are as follows:

**CuA** cubitus anterior

**CuP** cubitus posterior

**M** media

**R** radius

**RA** radius anterior

**RP** radius posterior

**ScP** subcosta posterior

**V** vannal

**Pcu** postcubitus

Measurements are based on specimens examined. The genital segments of the examined specimens were soaked in 10% NaOH and rinsed with distilled water, then stored in glycerin for observation. Genitalia were observed in glycerin using a MOTIC K400 stereomicroscope and genital photographs were taken using an LAS V4.9. Specimen photographs were taken using a Canon 50D plus a Canon EF 100mm f/2.8L IS USM MACRO lens and photos were stacked using Helicon Focus software. All photos were modified in Adobe Photoshop CS6. The materials examined are deposited in the Institute of Entomology, Southwest University, Chongqing (**SWU**) and the Shanghai Entomological Museum, the Chinese Academy of Sciences, Shanghai (**SHEM**).

## Taxonomy

### Allacta

Taxon classificationAnimaliaBlattodeaEctobiidae

Saussure & Zehntner, 1895

Allacta Saussure & Zehntner, 1895: 45 (New name for Abrodiaeta Brunner von Wattenwyl, 1893). Type species: Abrodiaetamodesta Brunner von Wattenwyl, 1893, by selection; Roth 1991: 996; Roth 1993: 361; Roth 1995: 51; Roth 1996: 235; Wang et al. 2014: 440.
Arublatta Bruijning, 1947: 224. Type species: Blattapunctata Walker, 1869. Synonymized by Roth 1991: 996.
Pseudochorisoblatta Bruijning, 1948: 90. Type species: Phyllodromiainterrupta Hanitsch, 1925, by selection. Synonymized by Princis 1965: 151.
Euhanitschia Princis, 1950: 178. Type species: Phyllodromiadiagrammatica Hanitsch, 1923. Synonymized by Roth 1996: 235.
Compsosilpha Princis, 1950: 180. Type species: Chorisoblattakarnyi Hanitsch, 1928. Synonymized by Roth 1996: 235.

#### Diagnosis.

Body pale brown to blackish brown, mostly yellowish brown. Clypeus normal, not thickened. Pronotum usually oval, wider than length. Tegmina and wings usually fully developed, a few slightly reduced (*T.hainanensis*). Tegmina with M, CuA oblique, hind wings with R straight, sometimes forked, M and CuA usually straight or nearly so, latter with 1–6 (usually 4 or 5) complete branches; apical triangle small, reduced or absent. Anteroventral margin of front femur Type B_2_ or B_3_; pulvilli present only on the fourth tarsomere of all legs, tarsal claws symmetrical and unspecialized, arolia present. Male abdominal terga unspecialized. Male genitalia usually have three principal phallomere sclerites, the hook-like phallomere on right side, the median, and the left; most species have an accessory median phallomere. Ootheca not rotated prior to deposition.

#### Note.

Roth (1996) and Wang et al. (2014) discussed the difference among *Allacta*, *Sundablatta*, and *Pseudophyllodromia*, but actually, *Allacta* is closely related with the three genera *Margattea*, *Balta*, and *Sorineuchora* according to Wang et al. (2017), which are also under the subfamily Pseudophyllodromiinae. The most striking difference between *Allacta* and its closely related genera is pulvilli only present on the fourth tarsomere of all legs; or anteroventral margin of front femur Type B_2_ or B_3_. It is possible that *Allacta* and *Balta* prove to be synonym in the future by molecular method.

#### Remarks.

Roth (1993) erected three species groups under *Allacta* based on color patterns and male interstylar margin, viz. *funebris* species group, *hamifera* species group, and *polygrapha* species group. According to Roth (1993), these three species can be put in the *hamifera* species group, *A.bruna* sp. n. by having a dark pronotum and keel-like ridge at interstylar margin (Figs 4, 11) and *A.alba* sp. n. and *A.hainanensis* (Liu et al. 2017) comb. n. by the two close lobes between the styli (Figs 23, 34).

##### Checklist of *Allacta* species from China

*Allactabimaculata* Bey-Bienko, 1969: 858. China (Yunnan, Guangxi)

*Allactaornata* Bey-Bienko, 1969: 859. China (Yunnan, Hainan)

*Allactarobusta* Bey-Bienko, 1969: 860. China (Yunnan)

*Allactatransversa* Bey-Bienko, 1969: 859. China (Hainan); Vietnam

*Allactaxizangensis* Wang et al., 2014: 449. China (Xizang)

*Allactaalba* sp. n. China (Zhejiang)

*Allactabruna* sp. n. China (Hainan)

*Allactahainanensis* (Liu et al., 2017), comb. n. China (Hainan)

##### Key to species of *Allacta* from China (males)

**Table:** 

1	Tegmina and wings reduced, not reaching the end of the abdomen	***A.hainanensis* (Liu et al., 2017), comb. n.**
–	Tegmina and hind wings fully developed, both extending beyond end of abdomen	**2**
2	Pronotal disc with trapezoidal symmetrical white maculae	***A.alba* sp. n.**
–	Pronotal disc without white maculae	**3**
3	Interocular space with pale brown horizontal stripe	**4**
–	Interocular space without pale brown horizontal stripe	**5**
4	Disc of pronotum totally blackish brown, subgenital plate asymmetrical	*** A. bimaculata ***
–	Disc of pronotum with irregular maculae, subgenital plate symmetrical	*** A. transversa ***
5	Pronotum brown without maculae	***A.bruna* sp. n.**
–	Pronotum with maculae	**6**
6	Head with 2 dark brown longitudinal stripes reaching from the vertex to the frons between the antennal sockets, and subgenital plate with dissimilar styli	*** A. robusta ***
–	Head with 1 dark brown longitudinal stripe reaching from the vertex to the clypeus or not, and subgenital plate with similar styli	**7**
7	Subgenital plate symmetrical, hind margin of subgenital plate nearly straight in the middle	*** A. ornata ***
–	Subgenital plate asymmetrical, hind margin of subgenital plate concave in the middle	*** A. xizangensis ***

### Allacta bruna sp. n.

Taxon classificationAnimaliaBlattodeaEctobiidae

http://zoobank.org/04466EA4-A061-436E-8872-6E50F80A2560

Figs 1–12 , 46

#### Type material.

***Holotype*** : male, CHINA, Hainan Province, Jianfengling, Mingfenggu, 960 m, 26-V-2014, Shun-Hua Gui, Xin-Ran Li et Jian-Yue Qiu leg. ***Paratypes***: CHINA, Hainan Province: 1 male, same data as holotype; 1 male, Limushan, 678–694 m, 17-IV-2015, Xin-Ran Li et Zhi-Wei Qiu leg.; 2 females, Jianfengling, Mingfenggu, 26-IV-2015, Lu Qiu et Qi-Kun Bai leg.; 1 female, Diaoluoshan, 17-IV-2015, Lu Qiu et Qi-Kun Bai leg. (all in SWU).

#### Diagnosis.

This species can be easily distinguished from its congeners by the brownish body that lacks any markings.

#### Measurements (mm).

Male, pronotum: length × width 4.5 × 6.8, tegmina length: 15.5, overall length: 18.9–19.2; female, pronotum: length × width 4.7 × 6.7–6.8, tegmina length: 14.1–14.3, overall length: 18.1–18.3.

#### Description.

**Figures 1–12.**
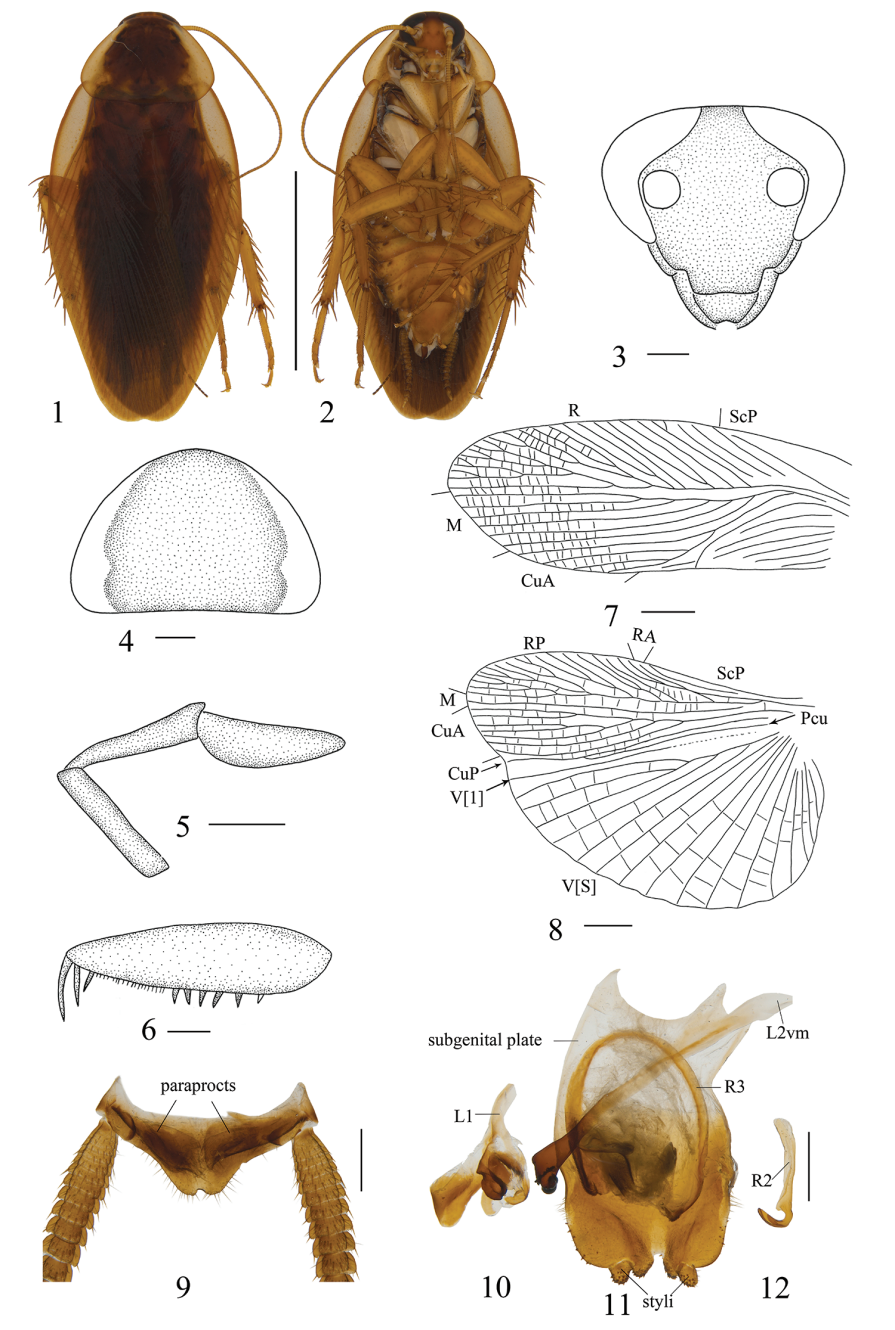
*Allactabruna* sp. n., male. **1** paratype, dorsal view **2** paratype, ventral view **3** head, ventral view **4** pronotum, dorsal view **5** maxillary palpi segments 3–5, ventral view **6** front femur, ventral view **7** tegmen, dorsal view **8** hind wing, dorsal view **9–12** holotype **9** supra-anal plate and paraprocts, dorsal view **10** left phallomere, dorsal view **11** subgenital plate and median phallomere, dorsal view **12** hook-like phallomere, dorsal view. Scale bars: 1.0 cm (**1, 2**), 0.5 mm (**3–8**), 1.0 mm (**9–12**).

***Male.*** Body brown to dark brown (Figs 1, 2). Head brown. Ocelli spots white. Antennae yellowish brown. Frons brown and clypeus pale brown. Maxillary palpi pale yellowish brown (Fig. 2). Pronotal disc dark brown, and two lateral borders yellowish brown (Figs 1, 4). Tegmina, wings, and legs brown (Figs 1, 2). Abdomen pale yellowish brown. Cerci black brown (Fig. 2).

Vertex with interocular space about half the distance between antennal sockets (Fig. 3). The third, fourth and fifth maxillary palpi of approximately same length (Fig. 5). Pronotum nearly trapezoidal, broader than long, maximum width behind the middle, the front and hind margins nearly straight, and the postero-lateral angle blunt and round (Fig. 4). Tegmina and wings fully developed, both extending beyond the end of abdomen. Tegmina with M and CuA longitudinal (Fig. 7). Hind wings with M straight and simple; CuA slightly curved with 4 branches (Fig. 8). Anteroventral margin of front femur type B_2_ (Fig. 6). Pulvilli only present on the fourth tarsomere. Tarsal claws symmetrical and unspecialized, arolium present. The first tarsus of the front legs about equal in length to the sum of the other four, but obviously longer in the middle and hind legs.

***Female*** similar to the male. The coloration of living individuals dark brown, dried specimens much lighter, especially in the legs. Margins of pronotum and tegmina subtransparent in the specimens.

***Male abdomen and genitalia.*** Abdominal terga unspecialized. Supra-anal plate nearly triangular, and the hind margin obviously concave. Paraprocts simple (Fig. 9). Subgenital plate nearly symmetrical, the middle part of the hind margin has a V-shaped concavity; styli similar, short cylindrical, with small spines, and interstylar margin with two lobes (Fig. 11). Left phallomere complex, inverted Y-shaped (Fig. 10). Median phallomere stem club-like, apex shoe-shaped, base with a small spine, the accessory structure curved, apex brush-shaped (Fig. 11). Hook on the right side (Fig. 12).

#### Remarks.

It can be distinguished from the other species in the group by the tegmina that without any light-colored bar in the anal field.

#### Etymology.

From the Latin word *brunus* referring to the body color being brown without any special markings.

#### Distribution.

China (Hainan).

### Allacta alba sp. n.

Taxon classificationAnimaliaBlattodeaEctobiidae

http://zoobank.org/290F2E8F-1E9D-42F0-9668-B18F9DA9A327

Figs 13–24

#### Type material.

***Holotype*** : male, CHINA, Zhejiang Province, Longtangshan, Qingliangfeng, 960 m, 6-VIII-2011, Jin-Jin Wang leg. ***Paratype***: 1 male, same data as holotype (all in SWU).

#### Diagnosis.

**Figures 13–24.**
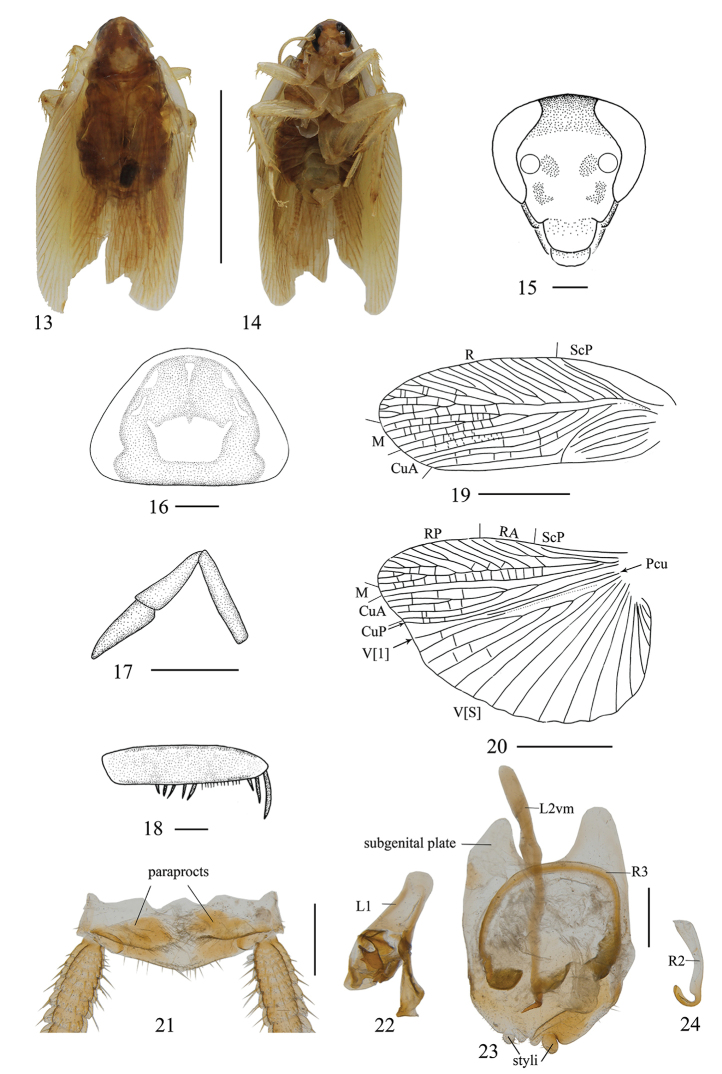
*Allactaalba* sp. n., male. **13** paratype, dorsal view **14** paratype, ventral view **15** head, ventral view **16** pronotum, dorsal view **17** maxillary palpi segments 3–5, ventral view **18** front femur, ventral view **19** tegmen, dorsal view **20** hind wing, dorsal view **21–24** holotype **21** supra-anal plate and paraprocts, dorsal view **22** left phallomere, dorsal view **23** subgenital plate and median phallomere, dorsal view **24** hook-like phallomere, dorsal view. Scale bars: 1.0 cm (**13, 14**), 0.5 mm (**15–20**), 1.0 mm (**21–24**).

This species resembles *A.xizangensis* by the brownish tegmina, which are without distinct strips and spots (Figs 13, 14, 44), and the two small lobes between the styli, but it can be differentiated from the latter by the following characters: 1) pronotum without dark brown strip-like markings, while with dark brown strip-like markings in *A.xizangensis*; 2) two styli unequal in size, while styli similar in *A.xizangensis*; 3) median phallomere with basal part tapering, while in the latter, blunt and round; and 4) R3 (accessary median phallomere) large, while R3 (accessary median phallomere) somewhat reduced in *A.xizangensis*.

#### Measurements (mm).

Male, pronotum: length × width 3.5–4.5, tegmina length: 14.1–14.8, overall length: 15.5–16.1.

#### Description.

***Male.*** Body yellowish brown with reddish (Figs 13, 14). Vertex brown. Face pale brown. Two spots on inter-antennae space reddish brown (Figs 14, 15). Antennae and maxillary palpi pale brown. Pronotal disc and hind margin reddish brown, lateral borders hyaline. Tegmina and wings pale yellow (Fig. 13). Legs translucent. Abdomen brown. Cerci yellowish brown (Fig. 14).

The distance between compound eyes narrower than that of antennae, ocelli border not obvious (Fig. 15). Curved macula under the antennal sockets. Third, fourth and the fifth maxillary palpi approximately same length (Fig. 17). Pronotum nearly triangular, width greater than length, the front and hind margins nearly straight, the postero-lateral angle blunt and round, and disc with trapezoidal symmetrical white maculae (Fig. 16). Tegmina and hind wings fully developed, both extending beyond the end of abdomen (Fig. 13). Tegmina with M and CuA longitudinal, CuA with four branches (Fig. 19). Hind wings with M straight and simple; CuA with five complete branches (Fig. 20). Anteroventral margin of front femur type B_3_ (Fig. 18). Pulvilli present only on the fourth tarsomere; tarsal claws symmetrical and unspecialized, arolia present.

***Male abdomen and genitalia.*** Abdominal terga unspecialized. Supra-anal plate nearly triangular, hind margin slightly curled up. Paraproct plates simple (Fig. 21). Subgenital plate slightly asymmetrical, styli short and cylindrical, the right one slightly larger than the left, inter-styli margin concave (Fig. 23). Left phallomere complex, inverted Y-shaped (Fig. 22). Median phallomere stem club-shaped, basal part tapering, accessory median sclerite curved, base and apex all with short setae (Fig. 23). Hook on the right side, with pre-apical incision (Fig. 24).

#### Remarks.

It can be distinguished from other species in this group by the light-colored pronotum.

#### Etymology.

The meaning of the Latin word *alba* is white, referring to the white maculae on the pronotum.

#### Distribution.

China (Zhejiang).

### Allacta hainanensis

Taxon classificationAnimaliaBlattodeaEctobiidae

(Liu et al., 2017) comb. n.

Figs 25–36 , 45

Temnopteryx hainanensis Liu et al., 2017: 179.

#### Type material examined.

***Holotype*** of *Temnopteryxhainanensis*, male (SHEM), CHINA, Hainan Province, Changjiang, Bawangling, 23–24-IX-2011, Xian-Wei Liu leg. ***Other material examined.*** CHINA, Hainan Province: 1 male and 2 females (SWU), Jianfengling, Mingfenggu, 23–28-IV-2015, Lu Qiu & Qi-Kun Bai leg. 1 male (SWU), Diaoluoshan, Lingshui, 1050m, 10-VIII-2010, Guo Zheng leg.

#### Diagnosis.

**Figures 25–36.**
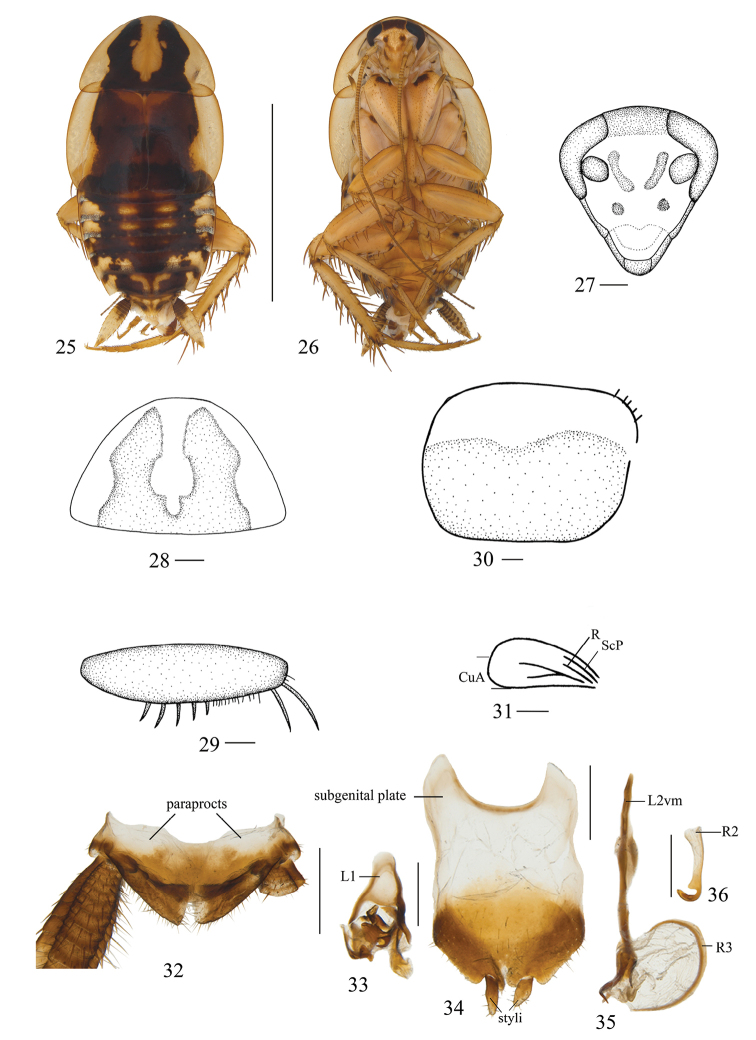
*Allactahainanensis* (Liu et al., 2017), comb. n., male. **25** dorsal view **26** ventral view **27** head, ventral view **28** pronotum, dorsal view **29** front femur, ventral view **30** tegmen, dorsal view **31** hind wing, dorsal view **32** supra-anal plate and paraprocts, dorsal view **33** left phallomere, dorsal view **34** subgenital plate, dorsal view **35** median phallomere, dorsal view **36** hook-like phallomere, dorsal view. Scale bars: 1.0 cm (**25, 26**), 0.5 mm (**27–31**), 1.0 mm (**32–36**).

This species can be easily distinguished from all other congeners by the much reduced tegmina and wings, which only reaching the third tergum. The coloration pattern (body light yellowish brown, with large brown areas dorsally) is also unusual in this genus (see Figs 25 and 45).

#### Measurements (mm).

Male, pronotum: length × width 4.3–4.6 × 6.9–7.4, tegmina length: 5.5–5.7, overall length: 17.0–17.2; female, pronotum: length × width 4.3–4.5 × 5.7–6.5, tegmina length: 4.6–4.9, overall length: 12.6–13.1.

#### Description.

***Male.*** Body dark brown with yellowish (Fig. 25). Frons yellowish brown, vertex with dark brown bands. Antenna brown. The fifth of maxillary palpomere brown (Fig. 26). Pronotum yellowish brown, lateral and hind border translucent, and disc with two dark brown stripes and an irregular pale yellowish brown macula. Tegmina brown, the inner border dark brown, lateral border pale yellowish brown (Fig. 25). Legs pale yellowish brown. Abdominal terga brown, lateral margin with blackish brown spots. Cerci yellowish brown, base brown (Fig. 26).

Vertex with interocular space obviously narrower than the distance between antennae sockets (Fig. 27). Third and fourth maxillary palpomeres approximately same length, and both significantly longer than the fifth. Pronotum nearly triangular, the front margin blunt and round; the hind margin nearly flat (Fig. 28). Tegmina and hind wings reduced; tegmina nearly quadrilateral, veins inconspicuous, only reaching the third tergum of the abdomen (Fig. 30); hind wings short and narrow, about half the length of the tegmina, and veins simple with two longitudinal veins (Fig. 31). Anteroventral margin of front femur type B_3_ (Fig. 29). Hind legs first tarsus approximately same length to the sum of other four tarsomeres. Pulvilli present only on the fourth tarsomere, tarsal claws symmetrical and unspecialized, arolia present (Figs 25, 26).

***Female*** similar to the male.

***Male abdomen and genitalia.*** Abdominal terga unspecialized. Supra-anal plate nearly triangular, symmetrical, the middle of hind margin with incisions. Paraproct plates simple, similar, sheet-like, apex with scattered bristles (Fig. 32). Subgenital plate symmetrical, lateral margin curved and blunt; styli nearly cylindrical, arising in two concavities of hind margin; the right stylus slightly longer than the left; and the hind margin with W-shaped concave (Fig. 34). Left phallomere complex (Fig. 33). Median phallomere stem slender, bending near apex, apex sharp, base forked, median phallomere subsidiary sclerite C-shaped clavate, apex gradually sharper (Fig. 35). The hook-shaped phallomere on the right of subgenital plate, and the hook short (Fig. 36).

#### Remarks.

Liu et al. (2017) placed this species in *Temnopteryx* and stated it resembles *T.dimidiatipes* Bolivar, 1890 from the Philippines. However, Princis (1957) erected *Lobopterella* and treated *T.dimidiatipes* as the type species. Thus *Lobopterelladimidiatipes* belongs to subfamily Blattellinae and does not display the characteristic of Pseudophyllodromiinae, viz. the hook-shaped phallomere on the right side (Roth 1988).

Through field efforts, we obtained several specimens with reduced tegmina and wings from Hainan Island. After comparing them with the type specimen (deposited in SHEM), we confirmed that our specimens are *Temnopteryxhainanensis* Liu et al., 2017. After dissecting the male genitalia, we found the hook-shaped phallomere of *Temnopteryxhainanensis* is on the right, and the pulvilli is present only on the fourth tarsomere, thus, *Temnopteryxhainanensis* should be placed in the pseudophyllodromiine genus *Allacta* as *Allactahainanensis* (Liu et al., 2017) comb. n.

This species is placed in the *hamifera* species group by having two lobes which forming a keel-like ridge, and in having the dark portion of the pronotum reduced to two longitudinal bands.

#### Distribution.

China (Hainan).

## Discussion

**Figures 37–42.**
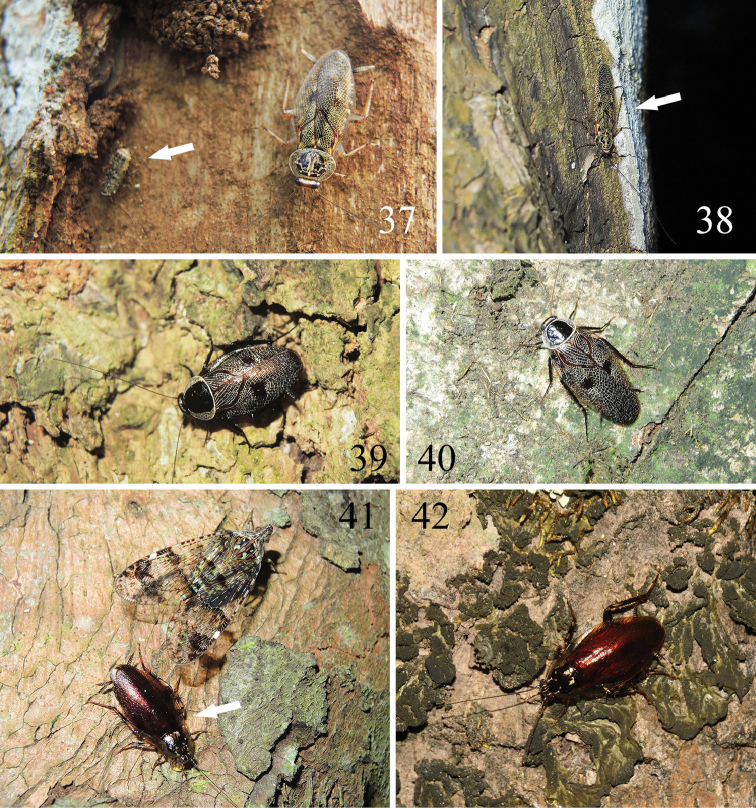
Habitats of *Allacta* species from China. **37, 38***Allactatransversa* from Hainan **37** when we remove the tree bark, a crowd of *A.transversa* run away and only an adult and a nymph (white arrow) hold still (Baoting County) **38** a very flat adult (white arrow) disguises itself in its surroundings (Baoting County) **39, 40***Allactabimaculata* from South China **39** a female resting on tree trunk (Longzhou, Guangxi) **40** a male resting on tree trunk (Xishuangbanna, Yunnan) **41, 42***Allactarobusta* from Pu’er, Yunnan **41** Individual (white arrow) found on the tree trunk along with a planthopper (Meizihu Lake, Simao) **42** Individual crawling on the tree trunk (Zhenyuan County). All photographs by Lu Qiu.

**Figures 43–46.**
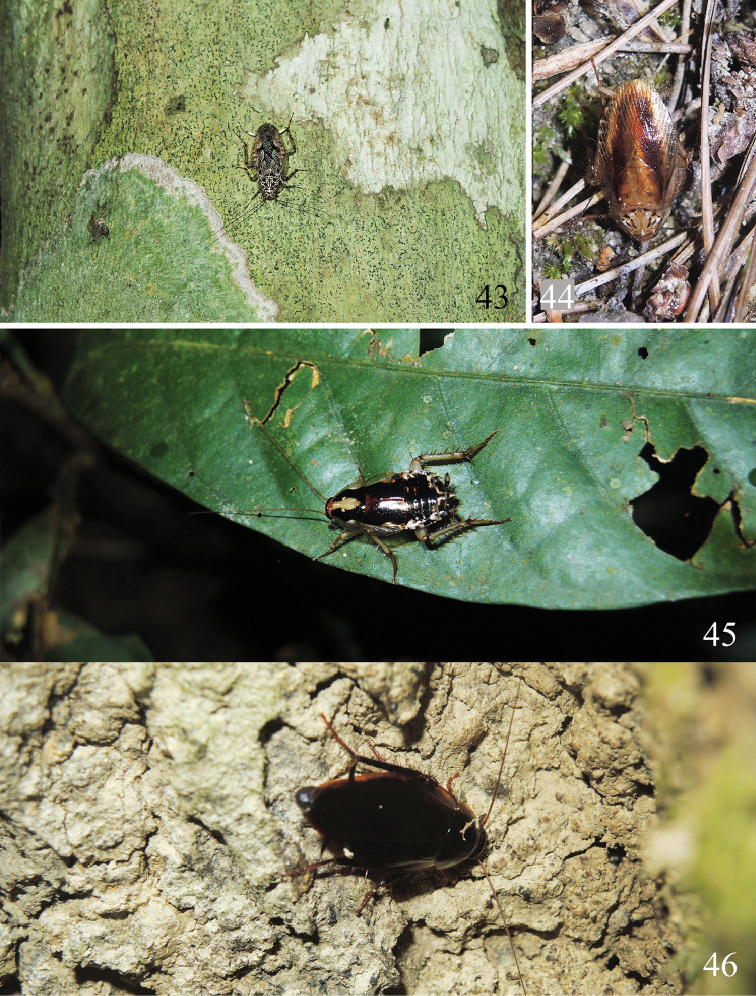
Habitats of *Allacta* species from China. **43***Allactaornata* found on tree trunk (Xishuangbanna, Yunnan) **44** most *Allactaxizangensis* were found on tree trunks during an expedition in VIII.2015, but this one is the only one found on the ground (Zayü County, Xizang) **45** a living *Allactahainanensis* (Liu et al., 2017) comb. n. was found on the leaf (Jianfengling, Hainan) **46** a female *Allactabruna* sp. n. (with ootheca) found on a tree trunk (Jianfengling, Hainan). All photographs by Lu Qiu.

Previous studies indicated that *Allacta* species inhabit on tree trunks (Rentz 2014; Wang et al. 2014). Through our field investigations from 2014 to 2017, we found that most Chinese *Allacta* species are more likely to inhabit tree trunk surfaces or under the bark. Sometimes, individuals are also observed crawling on the leaves (Fig. 45) or ground (Fig. 44). *A.bimaculata*, *A.ornata*, *A.robusta*, *A.transversa*, *A.bruna* sp. n., and *A.xizangensis* are observed resting on tree trunks. Their bodies are maculated (except for *A.bruna* sp. n.), and some species even have reticulated mottling on the tegmina (e.g., *A.bimaculata*, *A.ornata*, *A.transversa*), which provide an excellent disguise on a tree trunk surface that is covered with lichens and mosses (Figs 37–43). In the daytime, *Allacta* individuals may hide under the bark (Fig. 37), while during the night, they will come out and crawl on the trunk. When approaching them, they would not act dead or fall down to the ground, but instead hide from the collectors. They can move transversely somewhat like crab and very rapidly slip away to the opposite side of the trunk to escape from being captured. While when the collectors continue tracking them, they will move around to the other side of the trunk, or go to a higher level on the trunk. Characters such as the presence of pulvilli only on the fourth tarsomere and spines on the other 3 tarsomeres help *Allacta* species efficiently move about on the trunk. They can conduct such fast transverse actions probably because they only walk or climb “on tiptoe” (Wang et al. 2014).

## Supplementary Material

XML Treatment for Allacta

XML Treatment for Allacta bruna

XML Treatment for Allacta alba

XML Treatment for Allacta hainanensis
